# Psychometric validity of the parent’s outcome expectations for children’s television viewing (POETV) scale

**DOI:** 10.1186/1471-2458-14-894

**Published:** 2014-08-31

**Authors:** Teresia M O’Connor, Tzu-An Chen, Betty del Rio Rodriguez, Sheryl O Hughes

**Affiliations:** USDA/ARS Children’s Nutrition Research Center, Department of Pediatrics, Baylor College of Medicine, Houston, TX USA; Academic General Pediatrics, Department of Pediatrics, Baylor College of Medicine, Houston, TX USA

## Abstract

**Background:**

TV and other screen use are common among elementary school aged children with both potential benefits and harms. It is not clear why some parents restrict their children’s screen use and others do not. Parent’s outcome expectations for allowing their child to watch TV and other screen media, i.e. the perceived ‘costs’ and ‘benefits,’ may be influential. Our objective was to develop a measure of Parent’s Outcome Expectations for Children’s TV Viewing (POETV) and test the psychometrics of the resulting instrument among parents with children 6-12 years old.

**Methods:**

An ethnically diverse sample (n = 311) of parents from Harris County, Texas completed measures for POETV, demographics, and parent and child TV viewing and other screen media use via an internet survey. The sample was randomly split and an exploratory factor analysis (EFA) was conducted among the first half of the sample separately for Positive and Negative POETV. A confirmatory factor analysis (CFA) assessed the fit of the resulting factors with the data in the second half of the sample. Internal reliabilities and Spearman partial correlations (controlling for confounders) of children’s TV and other screen use with the resulting POETV factors were calculated for the full sample.

**Results:**

EFA identified two factors for Positive POETV (Parent Centered; Child Centered) and two factors for Negative POETV (TV & Content Exposure; Prevent Other Activities). Follow up CFA confirmed moderate to good psychometric properties for both factor structures with the addition of four correlated errors in the Positive POETV model. Internal reliabilities were appropriate (Cronbach’s alpha >0.7). Parent Centered Positive POETV and Child Centered Positive POETV were correlated with children’s TV viewing on weekdays (0.14, p < 0.05) and weekends (0.17, p < 0.01) respectively. Both also correlated with other screen media use on weekends (0.20 and 0.21, p < 0.001). Prevent Other Activities Negative POETV was negatively correlated with children’s TV viewing on weekdays (-0.16, p < 0.01), weekends (-0.14, p < 0.05) and other screen media on weekends (-0.14, p < 0.05).

**Conclusions:**

The Positive and Negative POETV scales offer a new tool to better define predictors of screen media parenting practices and child screen media use behaviors.

## Background

Television (TV) viewing is a common behavior among elementary school aged children and can have both potential negative and positive effects. Child TV viewing has been associated with overweight [1–3], aggressive behaviors [4], adolescents’ sexual attitudes and early initiation of sexual behaviors [5, 6], and worse school performance [7], especially if TV is viewed in early childhood [8]. Alternatively, viewing certain educational TV programs has been associated with improved vocabulary skills in preschool aged children [9] and reading achievement in school aged children [10]. Media interventions have also effectively promoted safe sex messages [11] and a CDC TV-commercial marketing campaign promoting physical activity (VERB™) had a dose effect on children’s free time physical activity and outcome expectancies [12].

Despite the potential benefits of TV viewing, the link between children’s TV viewing and obesity [1–3, 13–16] has become a public health concern. Children’s TV viewing may decrease their physical activity [3], increase their dietary intake [17–21], or both, resulting in excess energy balance. Given the potential enduring negative impact, with some potential positive effects of TV on youth, the American Academy of Pediatrics (AAP) recommended that children older than two years view no more than two hours of TV per day [4, 22]. Despite these recommendations, a third of US youth exceed 2 hours of TV viewing each day and almost half exceed two hours of total screen (TV and computer) time [23]. The home social [24, 25] and media environments [25–27] have been linked to children’s screen use. Children whose parents restricted their TV time viewed less television [28]. However, it is unclear why some parents restrict TV while others place little to no restrictions on their child’s TV viewing.

Parents’ outcome expectations of TV viewing for their child may impact their parenting regarding TV and thereby their child’s viewing time. Outcome expectations are an integral construct in Social Cognitive Theory [29]. They have been defined as the anticipated outcomes of actions and can be thought of as the pros (positive outcome expectations) and cons (negative outcome expectations) of engaging in a specific behavior [30]. Evidence suggests that parent’s outcome expectations affected their parenting and their child’s behaviors in contexts other than TV viewing [31, 32], but is not well studied for child behaviors regarding screen media use. For child TV viewing, which has both potentially positive and negative effects, it may be an important construct to explore. Associations among neighborhood characteristics [33], parental perception of neighborhood safety [34], and children’s TV viewing have been reported, suggesting that keeping a child safe in front of the TV (positive TV outcome expectations) may be more important for some parents than the child’s sedentary activity (negative TV outcome expectations), especially for those living in less safe neighborhoods.

Parents’ outcome expectations for letting their child view TV may need to be addressed for TV reduction interventions to be successful. For example, one intervention targeted social cognitive constructs to reduce preschool-aged children’s TV viewing with significant reduction in children’s TV viewing [35]. Parent’s outcome expectations demonstrated significant change in the intervention compared to control groups and was identified as the most useful social cognitive construct to target. However, the study employed a 7-item “convenience scale” developed for the study that focused on negative outcomes of TV viewing, since no existing validated scales existed in the literature [35]. No psychometrics of the scale were reported. This highlights the need to develop a measure of Parent’s Outcome Expectations of children’s TV Viewing (POETV) with factorial validity and appropriate reliabilities for use in future intervention and observational studies. Since parents report both positive and negative outcomes for letting their elementary school aged child view TV [36], both should be included in the instrument. The objective of this study was therefore to develop a measure of positive and negative POETV and test the factorial structure and psychometrics of the resulting instrument among parents with children 6-12 years old.

## Methods

### Development of the POETV Instrument

The POETV was developed based on semi-structured interviews with mostly Hispanic parents of self-reported overweight or obese 5-8 year old children regarding the reasons why children watch TV and reasons why parents may allow or limit TV viewing (36). Multiple themes emerged. Parents anticipated both positive outcomes of allowing their child to watch TV (e.g. a safe activity for children while parents get their work done, and fun entertainment for their child), and negative outcomes (e.g. affecting the child’s health, influencing family relations and exposing the child to inappropriate content) [36]. Based on the interviews, three of the authors developed 34 items that encompassed outcome expectations that the parents reported: 17 that were perceived as positive and 17 that were perceived as negative. Instructions to the instrument read: “How much do you agree or disagree with the following statements. If I let my child watch TV…” Response options were a 5-point Likert Scale (1- Strongly Disagree, 2-Disagree, 3-Not Sure, 4-Agree, and 5-Strongly Agree). The English version was translated into Spanish by a staff member fluent in Spanish, and independently back-translated into English by a second staff member to ensure content equivalence between the English and Spanish versions. Differences in the original and back-translated versions were reviewed and consensus was reached for conceptual and cultural rather than linguistic equivalence. Ten Hispanic parents participated in cognitive interviews while completing the 34 item POETV, 5 who completed the questionnaire in English and 5 who completed it in Spanish. There were no resulting changes in the POETV instructions, items or response options.

### Study design and sample

A cross-sectional multi-ethnic sample of parents with children 6-12 year old (n = 311) completed the study survey available on the internet. Inclusion criteria were 1) parent or legal guardian of a 6-12 year old child; 2) parent lives with the 6-12 year old child at least 50% of the time; 3) resides in Harris County, Texas (location of Houston); and 4) able to read and write in English or Spanish. Exclusion criteria included parent of a 6-12 year old child with medical or behavioral problems that prevents him/her from participating in age appropriate activities. The survey was developed on Survey Monkey GOLD software ©, such that the URL of the survey was SSL/HTTPS encrypted for data security. The online survey was available in English and Spanish.

Parents were recruited via flyers or short presentations introducing the study with the survey URL link, at community centers, community businesses (gyms, apartment complexes, restaurants, museums), WIC offices, libraries, pediatric clinics, the Texas Medical Center, universities and community college bulletins, health and food fairs, and the Children’s Nutrition Research Center (CNRC), Baylor College of Medicine (BCM), and Texas Children’s Hospital websites. Active CNRC research volunteers were informed of the study by email or phone. Interested parents accessed the online survey and informed online consent was obtained at the start of the survey with an introductory letter, followed by a set of pre-screening questions to assess eligibility. Only those parents who provided consent by clicking “I agree to participate” and met eligibility criteria could access the online survey. The survey was available on the internet from April 2012 to August 2012. The survey was accessed by 595 people, 486 agreed to participate, of whom 91 did not qualify per the screening protocol. Of the 395 who qualified, nine participants were removed from analyses because they completed the survey more than once, leaving 386 participants. This analysis included 311 parents (80.6% of those that accessed the survey and qualified) who completed the POETV portion of the survey, 94.2% who were mothers and reported mostly male children (57.9%). Participants had the option to opt into a drawing for one of 15 $100 gift cards as a thank you for participating. The demographic descriptors can be found in Table 1. The study was reviewed and approved by the Baylor College of Medicine Institutional Review Board.

**Table 1:** **Parent and child descriptive demographics and screen media use characteristics**

Variables	Statistic (n = 311)
**Parent/Child Characteristics**	
Parent gender, n(%)	
Female	293 (94.21)
Male	18 (5.79)
Parent age, mean(SD)	37.54 (8.39)
Child gender, n(%)	
Female	131 (42.12)
Male	180 (57.88)
Child age, mean(SD)	9.14 (2.42)
Parent education, n(%)	
<High School	20 (6.43 %)
High School/GED	34 (10.93 %)
Technical School/Some College	106 (34.09 %)
College Graduate	77 (24.76 %)
Graduate Study	74 (23.79 %)
Total household income, n(%)	
<=$19 k	31 (9.97 %)
$20 k-$49 k	98 (31.51 %)
> = 50 k	170 (54.66 %)
Unknown/No answer	12 (3.86 %)
Child Race, n(%)	
White	80 (25.72 %)
African-American	46 (14.79 %)
Hispanic	142 (45.66 %)
Other	43 (13.83 %)
**Child’s Screen use in hours**,^†^ mean (SD)	
Weekday TV & DVD viewing	2.39 (2.48)
Weekend TV & DVD viewing	3.95 (2.37)
Weekday all other screen media use	2.10 (2.78)
Weekend all other screen media use	3.18 (2.92)
**Parent’s Screen use in hours**,^†^ mean (SD)	
Weekday TV & DVD viewing	2.28 (1.96)
Weekend TV & DVD viewing	3.37 (2.3)
Weekday all other screen media use	2.06 (2.21)
Weekend all other screen media use	2.11 (2.02)

^†^Note: 3 participants reporting more than 20 hours TV viewing or all other screen media use were removed as they were outliers with are implausible values.

### Measures

In addition to the new POETV instrument, parents completed a demographic survey, and reported on their child’s screen media use. TV viewing by the child was assessed by parent report of 5 items that were a modified version of a global weekly TV viewing estimate that had reasonable correspondence to videotaped observations of children’s TV viewing in the home [37]. Each item was asked separately for weekdays and weekends to assess: 1) Watching TV on TVs, computers or other devices; 2) Watching videos or DVD’s; 3) Playing videogames (such as Xbox, Wii, Playstation); 4) Playing hand-held videogames (such as a DS, iPod or Leapster); 5) Using a computer for something other than activities related to school or work (such as browsing the internet, computer games, or e-mail). Parents responded by entering the average number of hours and minutes per day for each screen use question (or a ‘zero’ if child did not use that screen technology). For this analysis, we combined watching TV and videos or DVD’s into a “TV viewing” variable. Playing videogames, playing hand-held videogames, and using a computer for non-academic activities were combined into “other screen media” variable. Three outliers were removed from the TV viewing analysis who reported TV viewing or other screen media use for greater than 20 hours per day, which was implausible. Test-retest reliability of the TV and other screen media use from another unrelated study were 0.68 for TV viewing on weekdays, 0.89 for TV viewing on weekends,0.57 for other screen media use on weekdays, and 0.93 for other screen media use on weekends (O’Connor 2014 unpublished), suggesting moderate to high reliabilities.

### Analysis

The percentages, means and standard deviations were calculated for all demographic variables and screen viewing behaviors. A priori, we elected to assess the factor structure for Positive and Negative POETV in two separate models. Our qualitative data suggested that parents identified both positive and negative outcomes for allowing their child to view TV [6], suggesting that they are not opposite ends of the same construct, but two separate constructs. Attempting to assess the factor structure of a combined model, may therefore be overly complex. Similarly, other studies have identified separate models for effective and ineffective vegetable parenting practices [38, 39]. One item from the Positive POETV was dropped prior to analysis being run based on face validity (“…he/she would learn to speak better English.”), because it was deemed to only apply to a sub-section of the sample. This left 16 items for the Positive POETV factor analysis.

We randomly split our sample in two prior to running the factor analysis. Exploratory factor analyses (EFA) on the first half of the sample were conducted using principal components with a varimax rotation on Positive and Negative POETV items using scree plots to determine the number and structure of factors. Scree plot analysis suggested a 2 factor solution for both positive and negative POETV models. EFA was conducted using SPSS (Version 22.0, Armonk, NY: IBM Corp). Items which did not load on any factors (factor loading < 0.4) or loaded on more than one factor with factor loadings > 0.4, [40] were deleted from the scale and the EFA was rerun with the reduced set of items. This process was iterated until an optimal result was obtained. To evaluate the EFA model fit, items were submitted to confirmatory factor analyses (CFA) on the second half of the sample using Mplus (version 7.11, Los Angeles) with the Mean-adjusted Weighted Least Square (WLSM), which is appropriate for the evaluation of ordered categorical data. Model fit for the CFA was tested using Hu and Bentler’s two-index strategy [41], which includes the comparative fit index (CFI), the Tucker-Lewis index (TLI), the root mean square error of approximation (RMSEA), and the standardized root mean squared residual (SRMR). According to Hu and Bentler [41], good model fit should meet one of the following two-index criteria: (1) TLI ≥ 0.96 & SRMR ≤ 0.09; (2) RMSEA ≤ 0.06 & SRMR ≤ 0.09; or (3) CFI ≥ 0.96 & SRMR ≤ 0.09.

Internal consistency reliabilities (Cronbach’s alpha and mean inter-item correlations) were calculated. The acceptable Cronbach’s alpha, and mean inter-item correlation are values greater than 0.7 [42] and 0.2 [43], respectively. Spearman partial correlations (due to non-normal distributions) were calculated among Positive and Negative POETV factors; and for children’s weekday and weekend TV viewing and all other screen media use. The partial correlations controlled for child’s age, ethnicity and parent education, since these variables have previously been associated with child TV viewing. The internal consistency reliabilities and Spearman partial correlations was conducted in SAS 9.3 (New York 2011).

## Results

The EFA for the positive POETV identified two factors, with seven and five items each (Table 2). We dropped 4 items due to a non-substantial loading or a double loading. We labeled factor one “Parent Centered” because the items tended to reflect outcomes that helped the parent (e.g. “I would avoid arguments with him/her.” and “I would have time to socialize with others”) The second factor was called “Child Centered” because these items reflected a benefit to the child (e.g. “he/she would be entertained,” and “he/she would learn new things.”). The CFA confirmed good model fit after four correlated errors paths were added based on face validity and improved fit with the CFI ≥ 0.96 and SRMR ≤ 0.09(41) (Table 2).

**Table 2:** **Exploratory Factor loadings and Confirmatory Factor Model Fit Indices for Positive POETV (Parent’s Outcome Expectations for Children’s TV Viewing)**

If I let my child watch TV…	Factor loadings for Component	
	Parent Centered	Child Centered
05 …I would avoid arguments with him/her.	**.764**	-.071
29 …it would keep our home calm.	**.750**	.288
33 …he/she would not fight with family members (such as siblings).	**.731**	.104
23 …he/she would not bother me.	**.696**	.265
04 …he/she would calm down.	**.679**	.230
32 …I would have time to socialize with others.	**.635**	.108
03 …he/she would have something to do.	**.473**	.288
17 …he/she would be entertained.	.048	**.811**
25 …he/she would be occupied.	.317	**.742**
27 … he/she will not be bored.	.228	**.594**
08 …he/she would learn new things.	.003	**.577**
34 …we would have something to do together.	.205	**.537**
Dropped items		
01 …he/she would enjoy it.	-.229	.156
14 …I could relax.	.428	.635
20 …I would have time to do my work.	.433	.562
10 …he/she will be doing what other children his/her age do.	.090	.381
Chi-Square	236.676	
df	49	
p	<.001	
RMSEA	0.157	
CFI	0.957	
TLI	0.942	
SRMR	0.072	
Adding Paths	P05 WITH P04	
	P25 WITH P17	
	P33 WITH P32	
	P33 WITH P05	

Items 1, 14, 20 and 10 were excluded due to double loading or no substantial loading.

Bold factor loadings reflect the factor the item loaded onto.

The EFA for the negative POETV identified two factors with seven and six items each (Table 3). We dropped four items due to double loadings. We labeled factor one “TV and Content Exposure” because the items tended to reflect concepts that were associated with how the content or act of watching TV negatively impacts the child (e.g. “he/she would see too many inappropriate adult topics,” and “he/she would eat too many snacks.”). The second factor was called “Prevent Other Activities” because these items reflected the parents’ concerns about how TV watching would interfere with other activities or outcomes for the child (e.g. “we would have less time to spend together as a family,” and “he/she would not learn as many educational things.”). CFA confirmed good model fit with the CFI ≥ 0.96 and SRMR ≤ 0.09(41) (Table 3).

**Table 3:** **Exploratory Factor loadings and Confirmatory Factor Model Fit Indices for Negative POETV (Parent’s Outcome Expectations for Children’s TV Viewing)**

If I let my child watch TV…	Factor loadings for Component	
	TV and Content Exposure	Prevent Other Activities
31 …he/she would see too many inappropriate adult topics.	**.824**	.179
22 …he/she would see too much violence.	**.728**	.314
21 …he/she would learn unhealthy eating habits.	**.725**	.307
30 …he/she would gain weight.	**.697**	.248
28 …he/she would be exposed to too many food commercials.	**.672**	.301
09 …he/she would eat too many snacks.	**.607**	.168
02 …he/she would hear too much adult language.	**.581**	.061
06 …we would have less time to spend together as a family.	.143	**.773**
18 …it would be difficult to talk to him/her.	.162	**.771**
07 …he/she would be less active.	.105	**.710**
13 …he/she would not learn as many educational things.	.359	**.583**
19 …his/her speech would worsen.	.363	**.563**
24 …he/she would have less time to play outdoors.	.386	**.540**
Dropped items		
11 …his/her vision would get worse.	.381	.341
12 …he/she would not have time to do little house chores.	.635	.413
16 …his/her health would get worse.	.629	.450
26 …he/she would sit around too much.	.674	.404
Chi-Square	535.87	
df	64	
p	<.001	
RMSEA	0.218	
CFI	0.959	
TLI	0.95	
SRMR	0.08	

Items 11, 12, 16 and 26 were excluded due to no substantial loading or having double loading.

Bold factor loadings reflect the factor the item loaded onto.

All the factors had adequate internal consistency (Table 4). Spearman partial correlations (Table 4) showed that Parent-Centered Positive POETV was positively correlated with children’s TV viewing on weekdays (0.14, p < 0.05) and use of other screen media on weekends (0.20, p < 0.001). Child Centered Positive POETV was positively associated with children’s TV viewing on weekends (0.17, p < 0.01) and use of other screen media on weekends (0.21, p < 0.001). Prevent Other Activities, a Negative POETV, was negatively associated with children’s TV viewing on weekdays (-0.16, p < 0.01) and weekends (-0.14, p < 0.05) and all other screen use (-0.14, p < 0.05) on weekends. These correlations were all in the expected directions and support construct validity for the scales.

**Table 4:** **Mean summative factor scores, internal reliabilities and Spearman partial correlations** ^**§**^ **of Positive and Negative Parent Outcome Expectations for child TV viewing (POETV) with child TV viewing and use of other screen media**

	Mean (SD) Summative Scores*	Cronbach’s α	Mean Inter-item Correlation	Positive outcome expectations		Negative outcome expectations		Child TV viewing		Child use of other screen media	
				F1	F2	F1	F2	Weekday	Weekend	Weekday	Weekend
**Positive POETV**											
Parent Centered (F1)	17.11 (5.33)	0.81	0.39	1	0.56***	0.24***	0.22***	0.14*	0.1	0.1	0.20***
Child Centered (F2)	15.94 (3.75)	0.74	0.36		1	0.08	0.05	0.09	0.17**	0.11	0.21***
**Negative POETV**											
TV & Content Exposure (F1)	20.15 (6.53)	0.87	0.48			1	0.68***	-0.03	0.02	0.08	-0.01
Prevent Other Activities (F2)	17.68 (5.56)	0.83	0.45				1	-0.16**	-0.14*	-0.04	-0.14*

^§^Control for child’s age, parent education, and child ethnicity/race.

Child TV viewing: watching TV and videos or DVD’s.

Child Use of Other Screen Media: playing videogames, playing hand-held videogames, and using a computer for non-academic activities.

*Correlation significant at the 0.05 level (2-tailed).

**Correlation significant at the 0.01 level (2-tailed).

***Correlation significant at the 0.001 level (2-tailed).

## Discussion

Positive and Negative POETV scales were developed which had adequate factor structure fit and good internal reliabilities (all Cronbach alphas >0.7). Factorial validity of both the Positive and Negative POETV scales were demonstrated by meeting one of Hu and Bentler’s two-index criteria (CFI ≥ 0.96 & SRMR ≤ 0.09) [41]. Initial support for construct validity was demonstrated with correlations in expected directions with children’s TV viewing and other screen media use. Further construct validity will need to be established with future studies.

To date few studies have investigated theory-based predictors, likely to impact parental screen media restriction for their child or children’s screen media use. Identification of predictors, such as POETV, could prove useful as targets for future screen media reduction interventions. Previous research supports that parent’s attitudes, a type of outcome expectation, about the potential educational benefit of some TV programs was associated with the amount of TV viewed by children [44]. Greater perceived importance of screen media restriction was associated with greater restriction of children’s screen use among low-income parents of preschoolers [45]. Thus, parental attitudes and beliefs appear to influence their screen media parenting practices.

Only one other study has specifically investigated parental outcome expectations as a potential influence on children’s screen media use [35]. This TV reduction intervention highlighted that compared to parental self-efficacy and volitional control (which were not changed by the intervention) parental negative outcome expectation was an important attitude that could be changed and was associated with reductions in children’s TV viewing behaviors. The data presented here suggests that *both* Positive and Negative POETV separately and differentially influence children’s TV viewing. The POETV instrument described here may therefore prove to be an important tool that will allow a better understanding of how these separate constructs influence screen media parenting and ultimately children’s screen media use. To maximize impact, TV reduction interventions may need to not only increase parent’s Negative POETV but also reduce parent’s Positive POETV. For example, interventions may need to find alternative strategies other than TV or screen media for parents to keep their children occupied and homes calm.

It is noteworthy that the only POETV sub-scale not correlated with children’s TV viewing or other screen media use was TV and content exposure. The AAP not only recommends limiting the quantity of TV viewing and screen media use by children, but also endorses that parents carefully select appropriate content of TV programming viewed by children (4). In fact, the AAP encourages parents to co-view and discuss content with their child and teach critical viewing skills. While qualitative research supports that parents of elementary school aged children are concerned about the content that their children view [36, 46], it is not clear how this concern influences how they interact or parent their child regarding TV viewing and screen media use. Data presented here suggests that concern about the exposure of children to TV content is not correlated with how much TV their child views, but further research should investigate the role that parental concern about TV content has on rules regarding TV viewing and helping children select appropriate programming to watch.

This study had multiple strengths. The internet based sample was split in half which allowed for the factor structure to be explored in half the sample, and then confirmed it in the second half of the sample. Items were informed by parent input based on qualitative interviews. Participants included multiple ethnicities and represented diverse socio-economic backgrounds. Limitations of the study included a relatively small sample size (n = 311), an overrepresentation of relatively highly-educated parents, and parent report of children’s typical TV and other screen media use. Experts in the field were not consulted in this study to also generate items for positive and negative outcome expectations. Lastly, subjects were only recruited from one large metropolitan US city and Hispanics were overrepresented, all of which may limit its generalizability. However, the qualitative work used to develop the POETV instrument was also done in the same region and primarily with Hispanic parents [36] justifying the ethnic make-up of our sample for this first validation study. Future studies should allow experts to also generate other candidate outcome expectation items and confirm the factor structure and psychometric properties among other parent samples. The influence of the Positive and Negative POETV on parent’s use of restrictive and encouraging screen media parenting practices should be investigated.

## Conclusion

The Positive and Negative POETV scales show promising factorial validity, along with acceptable internal consistency reliabilities and initial support for construct validity. This new important tool can be used in observational studies and screen media reduction interventions aimed at assessing the role of parents on children’s screen media use and weight status.

## Abbreviations

TV: Television
AAP: American Academy of Pediatrics
POETV: Parent’s outcome expectations for children’s tv viewing
EFA: Exploratory factor analysis
CFA: Confirmatory factor analysis
CFI: Comparative fit index
TLI: The Tucker-Lewis index
RMSEA: Root mean square error of approximation
SRMR: Standardized root mean squared residual.
